# Peripheral Lymph Node Excisional Biopsy: Yield, Relevance, and Outcomes in a Remote Surgical Setup

**DOI:** 10.1155/2018/8120390

**Published:** 2018-03-20

**Authors:** Ashish Lal Shrestha, Pradita Shrestha

**Affiliations:** Department of General Surgery, United Mission Hospital, Tansen, Palpa, Nepal

## Abstract

**Objective:**

To study the patient profile for symptomatic peripheral lymphadenopathy in terms of histopathological findings and demography and evaluate the yield, relevance, and outcomes of peripheral lymph node biopsy (PLNB) as a diagnostic step in a remote setup in the absence of less invasive options like fine-needle aspiration cytology (FNAC) or ultrasonogram- (USG-) guided FNAC.

**Methods:**

A retrospective review of patients undergoing PLNB between 1 May 2011 and 30 April 2013 was done. Demographics, histopathological reports, and outcomes were studied.

**Results:**

Of 132 patients, 51 (38.63%) were male and 81 (61.36%) were female. There were 48 (36.3%) patients in the age group less than 16 years, and 84 (63.6%) were beyond 16 years. The commonest site of biopsy was the neck in 114 (86.36%) patients. The histopathological diagnosis was tuberculosis (TB) in 60 (45.45%) patients, reactive lymphadenitis in 29 (21.9%), nonspecific granuloma in 18 (13.6%), lymphoma in 7 (5.3%), acute lymphadenitis in 7 (5.3%), metastatic secondary in 3 (2.2%), and other benign causes in 8 (6.06%).

**Conclusions:**

PLNB is a procedure with good diagnostic yield in evaluation of peripheral lymphadenopathy. Its relevance is appreciable in a remote setup where less invasive options are unavailable. Its simplicity and lack of mortality/significant morbidity make it a valid option in rural surgical practice.

## 1. Introduction

Peripheral lymphadenopathy indicates any lymph node enlargement that is detectable on palpation at a location other than intrathoracic, mediastinal, intra-abdominal, or retroperitoneal [1]. In general, a node more than 1 cm or those smaller but multiple are considered abnormal [1–5]. The location is equally important. For instance, an isolated inguinal node can rarely be due to malignancy, while any palpable node in the supraclavicular, iliac, or popliteal regions can be suspicious [1, 2]. Likewise, the nodes even more than 5 mm if in the epitrochlear region are significant [6, 7]. Apart from the site and size, the consistency, the duration, and the rate of growth all are important [1, 2].

The retrospective reviews from the west suggest most of these to be self-limiting with biopsies yielding a malignant process only in the small group beyond 50 years [8]. A similar study reported 45% of cervical node biopsies to be benign and probably unnecessary [9]. More recent recommendations suggest the use of high-frequency ultrasound combined with FNAC (fine-needle aspiration cytology) in order to reduce the number of such biopsies [10, 11]. However, these findings need to be interpreted in light of situations where the prevalence of potentially curable infections like TB is high and other modern diagnostic facilities like high-frequency USG and FNACs are not available [3].

We studied the efficacy of open lymph nodal biopsy interpreting the results in view of rural population, the prevalent diseases, lack of modern facilities, and poor patient follow-up.

## 2. Methods

A retrospective analysis was done in the United Mission Hospital, Tansen, of rural western Nepal between 1 May 2011 and 30 April 2013. A total of 132 patients were enrolled following institutional approval. Relevant medical records of patients who underwent PLNB were reviewed. The following were the various indications for the procedure:

1. Significant localized or generalized peripheral lymphadenopathy lasting more than 2 weeks without a documented infectious cause
2. Lack of response to conservative treatment
3. Suspicion of a hematological malignancy
4. Suspicion of a secondary metastasis with occult primary cancer

The exclusions were made if the PLNB was not performed in isolation or was done as a part of a more elaborate procedure, for example:

1. Neck dissection for a known head and neck cancer with cervical lymphadenopathy
2. Axillary dissection for a known breast cancer with axillary lymphadenopathy
3. Inguinal block dissection for a known lower extremity malignancy
4. Intra-abdominal or retroperitoneal lymph node biopsy done for known malignancy

The results were analyzed using Microsoft Excel and SPSS Version 25.

## 3. Results

Of 132 patients, 51 (38.63%) were male and 81 (61.36%) were female. There were 48 (36.3%) patients belonging to the age group less than 16 years and 84 (63.6%) in the group above 16 years. Of these, children (<16 years) underwent the procedure under sedation, and those above 16 years tolerated the procedure well under local anesthesia. There was no event of perioperative complication. Postoperatively, pain was well controlled with oral analgesics, and all could be started on oral feeds and discharged the same day. Of these, one patient had a minor wound infection that was detected on the fifth postoperative day and treated with daily dressings and oral antibiotics, while none of the rest had any notable complications.

The most common site of biopsy was the neck in 114 (86.36%) patients as shown in Table 1.

In the age group >16 years, 59/84 (70%) had significant pathological yield, while in those <16 years, only 11/48 (23%) had significant findings as shown in Table 2. Using the chi-square test, the significance of this (59/84 versus 11/48) was calculated, and a *p* value of <0.001 was obtained that was considered highly significant.

Few benign, self-limiting diseases were also encountered in both the groups as listed in Table 3. Of these, the incidence of nonspecific granuloma (that did not stain positive for acid-fast bacilli) was 13.6% (18/132, with 9 in each group). These were all treated symptomatically. Similarly, the findings of sinus histiocytosis was comparable between both the groups, while cat-scratch disease and acute lymphadenitis were more common in adults.

**Figure 1:**
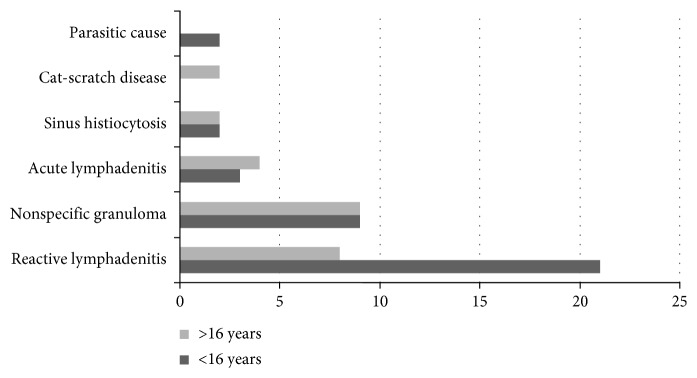
The findings of nonsignificant pathologies in the lymph nodal biopsies (*n*=62).

Lymphadenitis due to parasitic and reactive causes was clearly more in children as shown in Figure 1. All these benign causes were treated accordingly.

## 4. Discussion

A lymph node may be enlarged in certain situations, for example, an immune response to an infective agent (bacteria and virus), as a result of inflammatory cells in infections involving the lymph node (lymphadenitis), due to the infiltration of neoplastic cells carried to the node by lymphatic or blood circulation (metastasis), due to localized neoplastic proliferation of lymphocytes or macrophages (lymphomas), and as a result of infiltration of macrophages filled with metabolite deposits (lipid storage diseases) [8].

Peripheral lymphadenopathy may broadly be classified into generalized (when 2 or more noncontiguous areas are involved) or localized (when only 1 area is involved) [2].

While generalized lymphadenopathy is more concerning and almost always indicative of a significant systemic disease, it is the localized disease that presents with a bigger diagnostic challenge [2, 8]. It is often difficult to decide when to go ahead with a biopsy on a patient with unexplained peripheral lymphadenopathy.

In this regard, several studies have been done to reach to a sensible rationale of performing a PLNB. Many studies have tried developing a diagnostic algorithm with biopsy as the ultimate and last option, in order to avoid unnecessary biopsies especially when the yield of cancer has been reported to be fairly low [12].

These studies have suggested a histologically benign disease in 17–45% of cases in different sets of data [8–10]. In our study, we report cancer (lymphoma and metastatic secondaries) in only 7.5% cases. However, the findings of other benign but potentially treatable causes of lymphadenopathy like TB seem to be as high as 45.4%.

The yield of pathologically significant findings in our study was found to be much more when PLNB was performed in adult patients as compared to children (*p* < 0.001). The incidence of TB, primary lymphatic malignancy, and metastatic malignant deposits was similarly more with adult biopsies. Of these, the patients with TB were treated with antituberculous drugs after sputum tests and further workup, while the ones with primary lymphatic malignancy and malignant secondaries were referred to the regional cancer centre for further evaluation.

This seems to be in keeping with the high prevalence of TB in the community presenting still as a major disease burden and mortality. A recent study seems to follow a similar pattern, wherein the commonest diagnosis was TB (42%) [3].

While in the developed world, the practice has been to limit such biopsies much supported by the use of high-frequency USG (which can itself suggest diagnosis in expert hands) combined with FNAC and PLNB, which somehow seems to be imperative and unavoidable at various clinical situations in our setup [9–11].

The fact of relevance is that if an infectious workup is nondiagnostic and the patient has persistent or progressive lymphadenopathy of unknown cause, a biopsy is indicated [1], especially if the lymphadenopathy is in the supraclavicular or other cervical regions where the likelihood of finding a malignancy is higher compared to the other sites [2]. This should also be corroborated with other clinically relevant findings like background history, the size, progression, and consistency of nodes.

In our setup, we prefer to biopsy those patients who present with significant localized or generalized peripheral lymph nodes of more than 2- to 3-week duration without a documented infectious cause.

There are studies that quote a three- to four-week period of observation and some even up to 6 months [2, 8]. Due to lack of follow-up in our set of patients, we tend to biopsy them earlier. We biopsy such nodes after a thorough clinical and infectious workup and for indications mentioned above.

The incidence of a significant pathological diagnosis in our study was 53% (70/132), and this certainly demanded further evaluation. Had the biopsy not been done, the likelihood of missing these findings would be high, and hence the morbidity and mortality. In patients in whom the yield was insignificant, this could have caused psychological stress, but the overall end result can still be considered favorable in eliminating diagnostic doubt and dilemma. However, PLNB needs to be considered with caution in children in whom the likelihood of a pathologically significant yield seems less likely.

In our experience, we have found PLNB to be a relatively simple procedure.

Therefore, in view of its simplicity, good diagnostic yield, and lack of significant morbidity or mortality, in a rural setup like ours where expertise in terms of USG or FNACs is unavailable, it still seems to be a good diagnostic tool and an extremely useful procedure.

The limitations of our study include the bias associated with a retrospective observational study.

## 5. Conclusions

Peripheral lymphadenopathy is a common presentation in all age groups and often puts up a decisive dilemma regarding biopsy. A good clinical understanding can avoid an unnecessary biopsy in most cases, more so if facilities like USG combined with FNAC are available. However, these are not available to all.

With background knowledge of prevalent potentially curable diseases in the community like TB, it becomes desirable to consider this option when the patient follow-up is questionable. It is also advisable to biopsy the most abnormal node that may not necessarily be the most accessible one.

In view of simplicity, good diagnostic yield, and lack of significant morbidity or mortality, we conclude that open lymph node biopsy is an effective diagnostic tool in a peripheral setup.

## Figures and Tables

**Table 1:** Distribution of the lymph nodal biopsy site (*n*=132).

Lymph nodal site	Number of cases (%)
Neck	114 (86.36%)
Groin	11 (8.3%)
Axilla	7 (5.3%)
Total	132 (100%)

**Table 2:** Significant pathological yield.

Age group	TB lymphadenitis	Primary lymph nodal malignancy	Metastatic secondary	Total
<16 years	9	2	0	11
>16 years	51	5	3	59

**Table 3:** Distribution of diseases in biopsied lymph nodes (*n*=132).

Disease	Number of patients (%)
Tuberculosis	60 (45.4%)
Lymphoma	7 (5.3%)
Metastatic secondary	3 (2.2%)
Acute lymphadenitis	7 (5.3%)
Nonspecific granuloma	18 (13.6%)
Reactive lymphadenitis	29 (22%)
Other causes	8 (6%)
(i) Sinus histiocytosis	4 (3%)
(ii) Cat-scratch disease	2 (1.5%)
(iii) Parasitic cause	2 (1.5%)
Total	132 (100%)

## Abbreviations

USG: Ultrasonogram
TB: Tuberculosis
FNAC: Fine-needle aspiration cytology
PLNB: Peripheral lymph node biopsy.
